# Increasing use of sodium nitrite in suicides—an emerging trend

**DOI:** 10.1007/s12024-022-00471-8

**Published:** 2022-03-25

**Authors:** Lilli Stephenson, Stephen Wills, Corinna van den Heuvel, Melissa Humphries, Roger W. Byard

**Affiliations:** 1 Adelaide Medical School, The University of Adelaide, Level 2 Helen Mayo North, Frome Road, Adelaide, SA 5005 Australia; 2 School of Mathematical Sciences, The University of Adelaide, Adelaide, SA Australia; 3 Forensic Science South Australia (FSSA), Adelaide, Australia

**Keywords:** Suicide, Sodium nitrite, Methemoglobinemia, Increasing incidence

## Abstract

Sodium nitrite (NaNO_2_) is a compound with broad practical applications in food preservation, automotive maintenance, and animal control. Sodium nitrite is also potentially fatal when ingested, as it interferes with the ability of red blood cells to transport oxygen. While incidents of accidental consumption have been reported in the literature, case reports of sodium nitrite being used as a pharmacological suicide agent are becoming more frequent. Therefore, a study was undertaken to evaluate trends in sodium nitrite deaths in South Australia between 2000 and 2019. All cases of fatal sodium nitrite ingestion were identified in the Toxicology database and autopsy reports at Forensic Science SA (FSSA). Ten cases of fatal sodium nitrite ingestion were identified, with the rate of cases increasing significantly over the study period (*p* > .001). Of the ten deaths attributed to sodium nitrite toxicity, eight were male, and two were female. The age range of decedents was 22–74 years (mean 51.9, SD 21.1), 22–74 years for males (mean 58.4, SD 18.2) and 23–29 years for females (mean 26.0, SD 4.2). Males (80%) and those with a history of depression and/or suicidal ideation (70%) were over-represented within the study population. While autopsy findings were generally consistent with the literature, scene findings emphasized the accessibility of sodium nitrite to the general public. This study highlights both a significant increasing trend in the use of sodium nitrite in suicides and important analytical limitations in the evaluation of suspected cases.

**Supplementary information:**

The online version contains supplementary material available at 10.1007/s12024-022-00471-8.

## Introduction

Sodium nitrite (NaNO_2_) is a water-soluble, white-yellow-colored crystalline powder with various practical applications including use as a food preservative, antimicrobial, and coloring agent [1, 2]. It is also a corrosion inhibitor found in antifreeze [3, 4] and used as an antidote to cyanide poisoning [5]. In Australia, among other countries, it was also introduced as a method to humanely control the growth of feral pig populations [6]. Cases of accidental consumption in humans have been reported in the literature [7–18].

However, the use of sodium nitrite has recently emerged as an increasingly popular method of suicide. The first reported case of intentional sodium nitrite ingestion occurred in 1979 [19]. Then in 1990, a dental nurse ingested a 1 g sodium nitrite tablet obtained from her workplace [20]. Two cases were reported in Tokyo, Japan, one in 1996 and another in 2000 [21, 22]. Between 2000 and recent years, there has been a distinct lack of cases with only 1 case in New Zealand in 2010 identified in the literature [23]. However, since 2019 the number of reported suicides using sodium nitrite has significantly increased compared to previous years [24–32].

## Materials and methods

The database from the Toxicology Section at Forensic Science South Australia (FSSA) was searched for all post-mortem sodium nitrite detections over a 20-year period from January 2000 to December 2019, which were then matched against autopsy reports. Collected variables included age, sex, cause of death, location of death, scene findings, manner of death, autopsy, and toxicology findings.

Nitrate test strips designed for determining the presence of nitrate/nitrite in urine were used in the mortuary laboratory for the presumptive detection of nitrite/nitrate in the latter eight cases.

Routine toxicological analysis (alcohol and common drugs) was conducted at FSSA. However, FSSA does not currently have a validated method for the analysis of methemoglobin or nitrate/nitrite ions in post-mortem specimens. Therefore, post-mortem blood samples were sent to an external laboratory (SA Pathology, Royal Adelaide Hospital, North Terrace, Adelaide, South Australia) for methemoglobin analysis.

Statistical analyses were performed using R (version 4.1.2). A quasi-Poisson regression was used to characterize trends in the time series.

Ethics approval for the data used in this study was granted by the University of Adelaide Human Research Ethics Committee (H-2020–033).

## Results

### Rate of sodium nitrite suicides

**Fig. 1:**
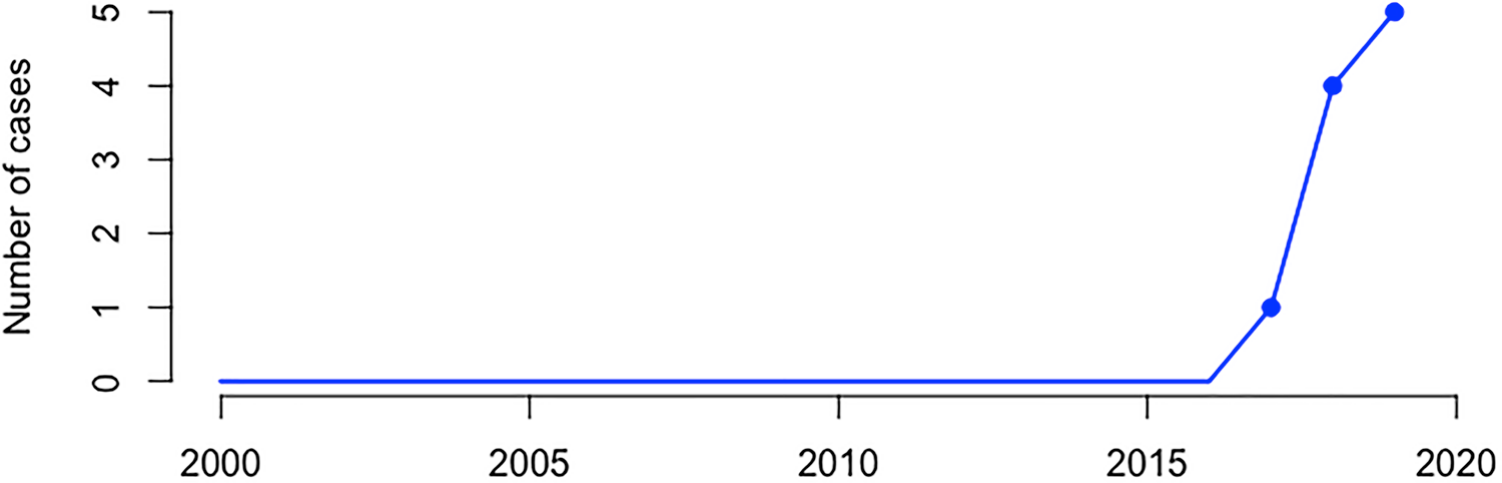
Numbers of sodium nitrite deaths in South Australia, Australia, between January 2000 and December 2019

Between 2000 and 2019, 10 cases were identified in which death had been attributed to methemoglobinemia due to sodium nitrite ingestion. All cases were a result of intentional ingestion, i.e., no cases of accidental exposure were identified. All 10 cases occurred in the latter 3 years of the study period with no sodium nitrite detections prior to 2017 (Fig. 1, see Appendix A for full statistical details). A quasi-Poisson regression confirmed a significant increase in the rate of sodium nitrite deaths over the study period (*p* > .001).

### Demographics

Of the 10 deaths attributed to sodium nitrite toxicity, eight were male, and two were female. The age range of all decedents was 22–74 years (mean 51.9, SD 21.1), 22–74 years for males (mean 58.4, SD 18.2) and 23–29 years for females (mean 26.0, SD 4.2).

### Medical history

Seven decedents had a medical history of depression, of whom four had expressed suicidal ideation. One decedent had a complex chronic medical history and had expressed his wish to be placed in palliative care and three who had previously attempted suicide including one previous attempt using sodium nitrite. While one decedent had no recorded history of depression, the medical histories of the two remaining decedents were unknown (Table 1).

**Table 1:** Sodium nitrite toxicity case findings

**Case no.**	**Age**	**Sex**	**History**	**Death scene**	**Autopsy**	Toxicology*	**External laboratory (SA pathology)**
1	74	M	• Hx: Emphysema, depression • Previous suicide attempt using sodium nitrite	• Empty bottle of sodium nitrite • Contacted ex-carer and neighbor to inform them of his intentions and say “goodbye”	• White, granular material in stomach contents • Blue-gray hypostasis • Brown discoloration of blood • Pulmonary edema	• Diazepam (0.02) • Nordiazepam (0.05) • Venlafaxine (0.75) • Desvenlafaxine (0.97) • Amlodipine (0.06) • Paracetamol (16) • Prochlorperazine (present)	• Sample was unsuitable for analysis
2	60	M	• Hx: Chronic neck pain and depression (10 years)	• A suicide note • 3 cans of Coca-Cola • Box of bicarbonate soda • 150 mL of clear liquid in a plastic jug • Piece of paper reading “10 g – 5 mL water” and “5 g sodium bicarb in water” • Empty packaging for “Back to Basics” homebrew supplies-brand sodium nitrite food grade (100 g)	• Blue-gray hypostasis • Brown discoloration of blood • Pulmonary edema	• Venlafaxine (0.9) • Desvenlafaxine (1.5) • Paracetamol (12) • Buprenorphine (0.6ug/L) • Metformin (0.63) • Mirtazapine (0.13) • COHb (31%)	• MetHb (87.5%) • COHb (negative)
3	74	M	• Resident at care facility • Hx: anxiety, chronic pain, depression, hypertension, lung carcinoma, neuropathic pain, osteoarthritis, previous prostate carcinoma… • Expressed desire to be in palliative care, previous reference to sodium nitrite as choice of method of suicide	• Suicide letters • Envelope containing an unsealed snap-lock bag labeled “sodium nitrite – food grade” from ‘back 2 basics artisan supplies’	• Blue-gray hypostasis • Pulmonary edema • Urine dipstick positive for nitrite • No evidence of ketosis or pathologically significant alteration of renal function	• Lorazepam (0.022) • Fentanyl (2ug/L) • Metoclopramide (0.14) • Pregabalin (4.0) • COHb (51%)	• Result was outside analytical limits^a^
4	69	M	• Relationship problems with wife • Hx: depression, haemochromatosis, hypertension, multiple myeloma, carpal tunnel syndrome…	• Empty bottle of wine • Small glass jar with two lines drawn in pen • Suicide note	• Blue-gray hypostasis • Brown discoloration of blood • Urine dipstick positive for nitrite • Pulmonary congestion	• Sertraline (0.32) • COHb (24%)	• Failed to give a valid result for methemoglobin due to specimen integrity
5	29	F	• Hx: depression, panic attacks, suicidal ideation • Previous suicide attempts and a hospital admission following overdose	• Called an ambulance after she had taken a mouthful of “sodium nitrate” • Farewell messages on phone • A Last Will and Testament document and a suicide note in an envelope • Container with an orange label “Sodium Nitrite” 50 g sourced from the Melbourne Food Ingredient Depot” with only a small amount remaining • A glass tumbler with white powder and liquid residue	• Blue-gray hypostasis • Brown discoloration of blood • Pulmonary edema and congestion • Urine dipstick positive for nitrite	• Naloxone (present) • COHb (25%)	• The sample failed to give a valid result on the analyzer
6	64	M	• Hx: previous colon cancer, diverticular disease, chronic back and leg pain, gastric reflux	• 2 × white powder (sodium nitrite, sodium bicarbonate) • Clear, colorless liquid (unknown) • Pale, yellow powder (sodium nitrite)	• Blue-gray hypostasis • Brown discoloration of blood • Pulmonary edema • Terminal aspiration of crystalline birefringent/refractile material • Urine dipstick positive for nitrite	• Paracetamol (24) • Codeine (0.08) • Prochlorperazine (present) • Ranitidine (present) • Loperamide (present) • COHb (34%)	• Methemoglobin analysis available was unable to produce a methemoglobin result
7	23	F	• Hx: bipolar disorder, possible borderline personality disorder, suicidal ideation	• Open bottle of sodium nitrite • Two drinking glasses, one half-full containing a yellow transparent substance and powder residue at the bottom • Suicide note • Mobile search history to “sanctionedsuicides.com” regarding methods of suicide including references to sodium nitrite	• Blue-gray hypostasis • Brown discoloration of blood • Moderate pulmonary edema and congestion • Urine dipstick positive for nitrite	• Metoclopramide (0.83) • Quetiapine (1.6) • Paracetamol (100) • COHb (51%)	• Due to sample clotting, unable to process for methemoglobin testing
8	64	M	• Hx: Type 2 diabetes mellitus, hypercholesterolemia, depression • Granted home detention bail	• Suicide notes • A glass with white residue • Plastic bag containing sodium nitrite located in desk drawer • Cylinders of nitrogen gas found under desk and in vehicle	• Early putrefactive changes • Blue-gray hypostasis • Brown discoloration of blood • Pulmonary edema and congestion • Urine dipstick positive for nitrite	• Alcohol (0.038%) • Metoclopramide (0.21) • Paracetamol (32) • Zolpidem (present) • COHb (40%)	• Sample received clotted and unable to be analyzed for methemoglobin
9	22	M	• Hx: unknown	• Clear substance in bottle next to deceased • Suicide-type note, papers and receipts	• Blue-gray hypostasis • Brown discoloration of blood • Urine dipstick positive for nitrite	• Metoclopramide (0.2) • Ranitidine (present) • Paracetamol (19) • COHb (24%)	• Instrument was unable to process the sample due to viscosity
10	40	M	• Hx: unknown	• Sodium nitrite suicide kit • Suicide note	• Advanced putrefactive change • Urine dipstick positive for nitrite	• Alcohol (0.073%)^b^ • Paracetamol (33)^b^ • Metoclopramide (0.62)^b^ • Paracetamol (90 mg/kg)^c^ • Metoclopramide (2 mg/kg)^c^	N/A

^*^mg/L unless stated otherwise

^a^Measurable range is 120–180 g/L

^b^Putrefactive effusion

^c^Liver

### Scene findings

In all cases, evidence of sodium nitrite ingestion was found at the death scene (e.g., labeled sodium nitrite packaging, drinking glasses, and white powder residue). However, the source of sodium nitrite could only be identified in four cases. Food-grade sodium nitrite was sourced from a homebrew supply in two cases and from a food store in another. In one case, a sodium nitrite suicide kit was sourced via the post. Unfortunately, the source of sodium nitrite in the remaining cases was not recorded or could not be ascertained. A suicide note(s) was found at the scene in all but two cases, with a verbal expression of intent to commit suicide in one additional case (Table 1).

### Autopsy findings

**Fig. 2:**
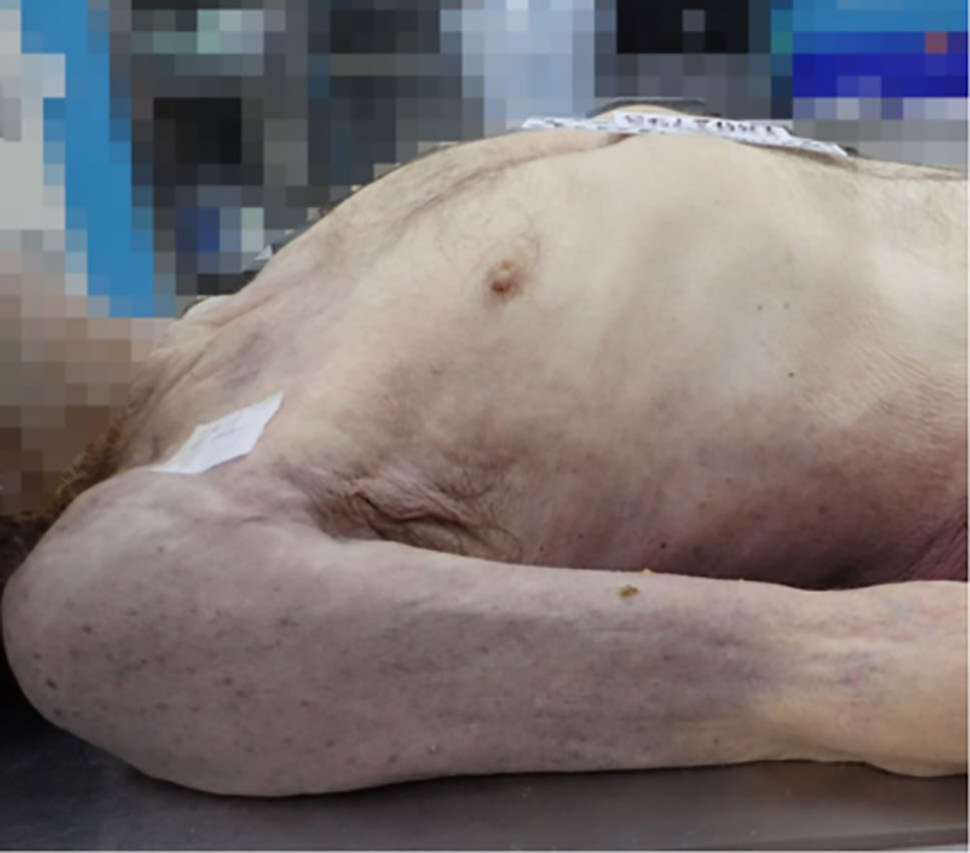
Characteristic gray-blue discoloration of the skin in a case of lethal sodium nitrite toxicity

**Fig. 3:**
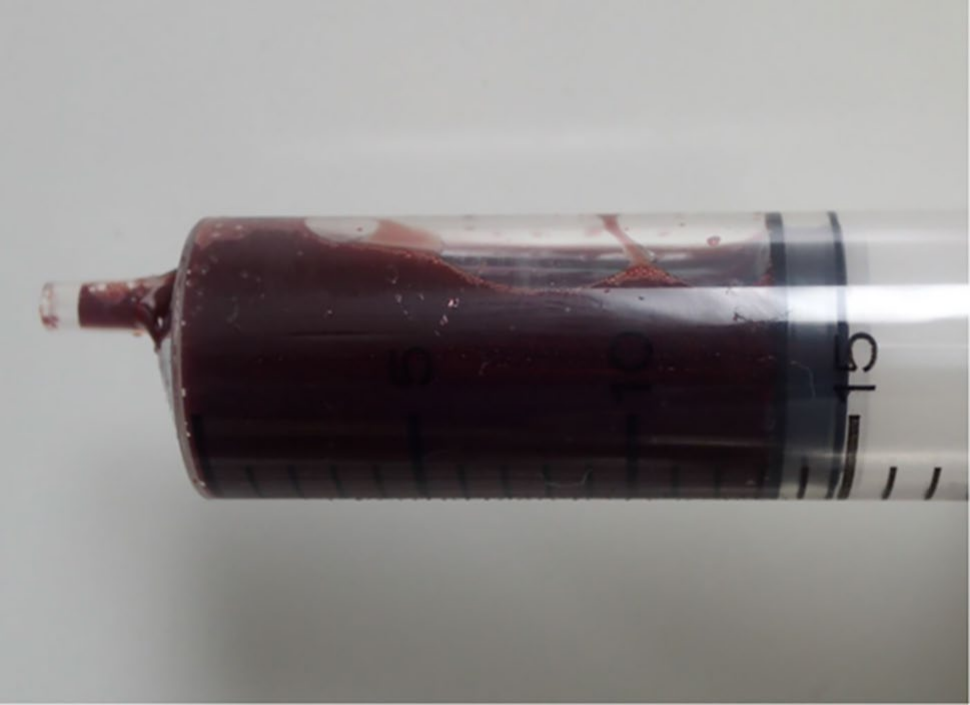
Characteristic red-brown discoloration of blood in a case of lethal sodium nitrite toxicity

Autopsy examination revealed findings consistent with post-mortem signs of methemoglobinemia including blue-gray hypostasis (Fig. 2) and dark brown discoloration of the blood (Fig. 3) and internal organs in all but one case. In the latter, advanced decomposition impaired identification of characteristic post-mortem features.

**Fig. 4:**
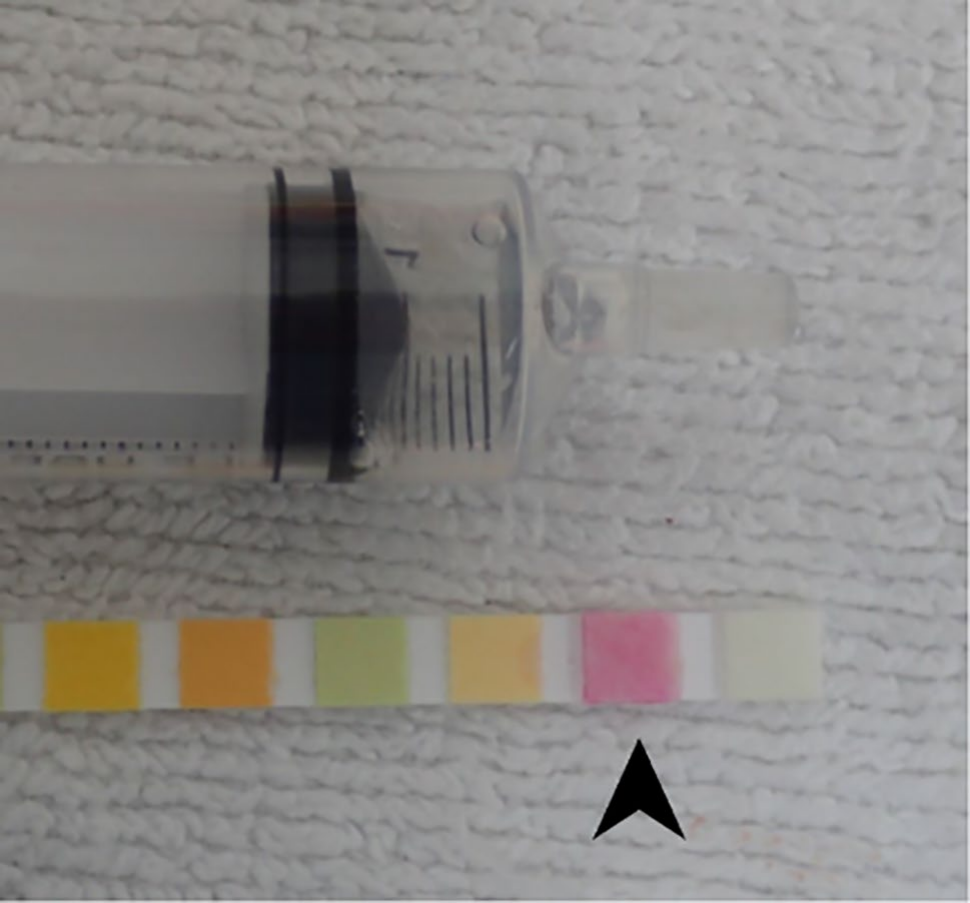
Positive nitrite result of a urinalysis dipstick in a case of lethal sodium nitrite toxicity

Although not a validated analytical method for this application, presumptive testing was performed in the most recent eight cases using a urinalysis dipstick (Fig. 4) on urine, vitreous humor, and/or gastric contents, all of which returned a positive nitrite result (Table 1). There was no indication that any of the decedents had a urinary tract infection.

In each case, no underlying organic diseases or injuries were identified that could have caused or contributed to death.

### Toxicology findings

At present, FSSA does not have a validated analytical method for nitrite or methemoglobin in body fluids. It should be noted that methemoglobin presence in post-mortem blood can interfere with some reductive-spectroscopic methods for determination of carboxyhemoglobin (COHb). This led to initial return of false COHb readings in several samples.

Most cases also demonstrated the presence of other drugs in the post-mortem blood sample that were either within the therapeutic range or related to resuscitation attempts (Table 1).

### External laboratory findings (SA pathology)

Post-mortem blood samples were submitted to an external laboratory for analysis of methemoglobin. Although one case showed 87.5% methemoglobin in the blood, the post-mortem blood samples were deemed either unsuitable for analysis or were outside the established analytical range in the remaining cases (Table 1).

Thus, the finding of sodium nitrite toxicity as the cause of death was based upon scene findings and characteristic autopsy findings rather than toxicological confirmation due to an inability to unequivocally confirm the presence of nitrite, nitrate, or methemoglobin in most cases.

## Discussion

The use of sodium nitrite as a suicide method is recommended by *The Peaceful Pill Handbook* [33]. Additional information and “suicide kits” may also be obtained from online forums and websites [26], with an example of this identified in the current study. Sodium nitrite is convenient to use as an agent in suicide as it is inexpensive, widely available, and easy to use; i.e., it can simply be taken as a drink when dissolved in water. The lethal dose of sodium nitrite has been estimated to be between 1 g and 2.6 g [24, 27], whereas *The Peaceful Pill Handbook* recommends a 15 g dose [33]. However, there have been cases where patients have survived doses exceeding 15 g with prompt medical care [24, 34].

Sodium nitrite acts by interfering with red blood cells binding to oxygen. The iron component of hemoglobin becomes oxidized from ferrous iron (Fe^2+^) to ferric iron (Fe^3+^), converting hemoglobin to methemoglobin [1]. Methemoglobin cannot bind oxygen which results in impaired oxygen transport, subsequent hypoxia, and lactic acidosis. Circulatory dysfunction is further compounded by hemolysis and the peripheral vasodilatory action of sodium nitrite (as a precursor of nitric oxide), inducing circulatory shock [35, 36]. As such, pre-existing cardiovascular disease and/or anemia are conditions which will exacerbate toxicity [37]. The physical symptoms of sodium nitrite poisoning vary depending on the concentration of methemoglobin but first become evident at levels of approximately 35% and include fatigue, difficulty/irregular breathing, tachycardia, impaired mental status, nausea, and vomiting [28]. At above 50%, patients develop symptoms of severe tissue hypoxia such as cardiac arrhythmias, seizures, coma, and death [38, 39].

Historically, documented cases of sodium nitrite toxicity were almost exclusively caused by inadvertent ingestion. While sodium nitrite suicides are still relatively rare, the rapid increase in suicidal cases during the latter 3 years of the study period with no cases prior to this is cause for concern. A similar trend has been reported in recent years in the United States (US) [24, 28], Portugal [26, 27], and the Republic of Korea [40]. It is highly likely that this trend will plateau over future years rather than continuing at the same rate. However, further monitoring is important to determine the trajectory of such trends and whether intervention is required.

It is difficult to determine the reasons for this increase with certainty. However, it is possible that the recent online availability of publications such as the *Peaceful Pill eHandbook* has disseminated information to a larger, online audience. It contains a chapter on Lethal Inorganic Salts, including sodium nitrite, among others [41]. The emergence of inert gas inhalation as a method of suicide was also thought to be influenced by the increasing availability of information on the internet [42]. Sodium nitrite is also cheap and easy to obtain with two decedents having purchased food-grade sodium nitrite from a homebrew supplier in the current study.

For the cases presented, diagnosis of sodium nitrite toxicity was based on a wide variety of information obtained from the decedent history, death scene, autopsy, toxicology, and biochemistry findings, often in the absence of a definitive, quantitative diagnostic test. While there are several other possible causes of methemoglobinemia, both congenital and acquired (including recessive congenital methemoglobinemia [43], mothball intoxication [44] and dapsone overdose [45], and several other substances [46]), all death investigations demonstrated evidence of sodium nitrite ingestion. Furthermore, use of the urinalysis dipstick for presumptive nitrite detection was applied in the latter eight cases, which all returned a positive result, raising suspicion of potential sodium nitrite toxicity and prompted further investigations. While unvalidated for such an application, the positive predictive value for detection of nitrite using a urinalysis dipstick in the diagnosis of urinary tract infection has been shown to be highly reliable, although slightly less so in men (86% and 63%, respectively), with an assumed sensitivity of 45% and specificity of 85% overall [47]. In addition to a presumptive positive nitrite urinalysis result, diagnosis of fatal sodium nitrite toxicity was made based on scene findings (e.g., sodium nitrite packaging, powder, liquid, suicide note), medical history (e.g., depression, suicidal ideation, previous suicide attempts), autopsy findings (e.g., blue/gray hypostasis, brown discoloration of blood and internal organs) toxicology, and external laboratory findings.

Awareness of emerging fatal substance abuse trends, clinical symptoms, and post-mortem signs by health professionals and pathologists is critical to effectively identify these cases at presentation and autopsy. This is particularly the case where routine toxicological methods may not be available. This study also highlights the potential usefulness of presumptive qualitative (albeit unvalidated) testing for sodium nitrite using urinalysis dipsticks in suspected cases of sodium nitrite overdose, particularly where blood sample analysis may not yield meaningful results.

## Key points

1. Sodium nitrite is cheap, widely available, and easy to use as a suicidal agent.
2. A rapid increase in sodium nitrite suicides has been identified in the South Australian autopsy population between 2000 and 2019.
3. Adult males with a history of depression and/or suicidal ideation were over-represented in the study population.
4. There are significant analytical limitations associated with the evaluation of methemoglobinemia in post-mortem samples.

## Supplementary information

Below is the link to the electronic supplementary material.

Supplementary file1 (DOCX 13 KB)

## References

[1] Benowitz N, Olson K (2018). Nitrates and nitrites. Poisoning and drug overdose.

[2] Laue W, Thiemann M, Scheibler E, Wiegand KW. Nitrates and nitrites. Ullmann's Encyclopedia of Industrial Chemistry. 2000.

[3] Abohalguma T, Elshawesh F, Elahresh N, BinZiglam W. Effect of pH on the performance of sodium nitrite corrosion inhibitor. EUROCORR 2004 - European Corrosion Conference: Long Term Prediction and Modelling of Corrosion. 2004.

[4] Farkas AN, Scoccimarro A, Pizon AF (2017). Methemoglobinemia due to antifreeze ingestion. N Engl J Med.

[5] Bebarta VS, Brittain M, Chan A, Garrett N, Yoon D, Burney T, Mukai D, Babin M, Pilz RB, Mahon SB, Brenner M, Boss GR (2017). Sodium nitrite and sodium thiosulfate are effective against acute cyanide poisoning when administered by intramuscular injection. Ann Emerg Med.

[6] Shapiro L, Eason C, Bunt C, Hix S, Aylett P, MacMorran D (2016). Efficacy of encapsulated sodium nitrite as a new tool for feral pig management. J Pest Sci.

[7] Gautami S, Rao RN, Raghuram TC, Rajagopalan S, Bhat RV (1995). Accidental acute fatal sodium nitrite poisoning. J Toxicol Clin Toxicol.

[8] Centers for Disease Control and Prevention. Methemoglobinemia following unintentional ingestion of sodium nitrite-New York, 2002. MMWR Morb Mortal Wkly Rep. 2002;51:639–42. 12186221

[9] Ellis M, Hiss Y, Shenkman L (1992). Fatal methemoglobinemia caused by inadvertent contamination of a laxative solution with sodium nitrite. Isr J Med Sci.

[10] Cvetković D, Živković V, Lukić V, Nikolić S (2019). Sodium nitrite food poisoning in one family. Forensic Sci Med Pathol.

[11] Bradberry SM, Gazzard B, Vale JA (1994). Methemoglobinemia caused by the accidental contamination of drinking water with sodium nitrite. J Toxicol Clin Toxicol.

[12] Lee C, Jang EJ, Yum H, Choi YS, Hong J (2017). Unintentional mass sodium nitrite poisoning with a fatality. Clin Toxicol (Phila).

[13] Kennedy N, Smith CP, McWhinney P (1997). Faulty sausage production causing methaemoglobinaemia. Arch Dis Child.

[14] Jiranantakan T, Olson KR, Tsutaoka B, Smollin CG (2016). Methemoglobinemia from frozen-dried mudfish contaminated with sodium nitrite. Clin Toxicol (Phila).

[15] Maric P, Ali SS, Heron LG, Rosenfeld D, Greenwood M (2008). Methaemoglobinaemia following ingestion of a commonly available food additive. Med J Aust.

[16] Ten Brink WA, Wiezer JH, Luijpen AF, Van Heijst AN, Pikaar SA, Seldenrijk R (1982). Nitrite poisoning caused by food contaminated with cooling fluid. J Toxicol Clin Toxicol.

[17] Aquanno JJ, Chan KM, Dietzler DN (1981). Accidental poisoning of two laboratory technologists with sodium nitrite. Clin Chem.

[18] Freeman L, Wolford RW. Methemoglobinemia secondary to cleaning solution ingestion. J Emerg Med. 1996;14:599–601. https://www.sciencedirect.com/science/article/pii/S0736467996001394. 10.1016/s0736-4679(96)00139-4 8933322

[19] Standefer JC, Jones AM, Street E, Inserra R (1979). Death associated with nitrite ingestion: report of a case. J Forensic Sci.

[20] Gowans WJ. Fatal methaemoglobinaemia in a dental nurse. A case of sodium nitrite poisoning. Br J Gen Pract. 1990;40:470–1. PMC1371420 2271282

[21] Saito T, Takeichi S, Yukawa N, Osawa M (1996). Fatal methemoglobinemia caused by liniment solutions containing sodium nitrite. J Forensic Sci.

[22] Saito T, Takeichi S, Osawa M, Yukawa N, Huang X-L (2000). A case of fatal methemoglobinemia of unknown origin but presumably due to ingestion of nitrate. Int J Legal Med.

[23] Harvey M, Cave G, Chanwai G. Fatal methaemoglobinaemia induced by self-poisoning with sodium nitrite. Emerg Med Australas. 2010;22:463–5. https://onlinelibrary.wiley.com/doi/abs/10.1111/j.1742-6723.2010.01335.x. 10.1111/j.1742-6723.2010.01335.x 21040485

[24] Mudan A, Repplinger D, Lebin J, Lewis J, Vohra R, Smollin C. Severe methemoglobinemia and death from intentional sodium nitrite ingestions. J Emerg Med. 2020;59:e85-e8. https://www.sciencedirect.com/science/article/pii/S0736467920305801. 10.1016/j.jemermed.2020.06.031 32713620

[25] Workum JD, Bisschops LLA, van den Berg MJW. [Autointoxication with 'suicide powder']. Ned Tijdschr Geneeskd. 2019;163. 30875162

[26] Durão C, Pedrosa F, Dinis-Oliveira RJ. A fatal case by a suicide kit containing sodium nitrite ordered on the internet. J Forensic Leg Med. 2020;73:101989. 10.1016/j.jflm.2020.101989 32658747

[27] Durão C, Pedrosa F, Dinis-Oliveira RJ (2020). Another suicide by sodium nitrite and multiple drugs: an alarming trend for “exit”? Forensic Science. Medicine and Pathology.

[28] Dean DE, Looman KB, Topmiller RG. Fatal methemoglobinemia in three suicidal sodium nitrite poisonings. J Forensic Sci. 2021. 10.1111/1556-4029.14689 33598944

[29] Sedhai YR, Atreya A, Basnyat S, Phuyal P, Pokhrel S. The use of sodium nitrite for deliberate self-harm, and the online suicide market: should we care? Med Leg J. 2021:25817221998119. 10.1177/0025817221998119 33906496

[30] Tomsia M, Głaz M, Nowicka J, Szczepański M. Sodium nitrite detection in costal cartilage and vitreous humor - case report of fatal poisoning with sodium nitrite. J Forensic Leg Med. 2021;81:102186. 10.1016/j.jflm.2021.102186 34058704

[31] McCann SD, Kennedy JM, Tweet MS, Bryant SM (2021). Sodium nitrite ingestion: an emerging trend in suicide attempts shared via online communities. J Emerg Med.

[32] Barranco R, Frigiolini FME, Orcioni GF, Malandrino M, Salomone A, Ventura F (2021). A rare case of fatal self-poisoning with sodium nitrite: autopsy and toxicological findings. Am J Forensic Med Pathol.

[33] Nitschke P, Stewart F. Lethal inorganic salts. The Peaceful Pill Handbook. Blaine: Exit International USA. 2018;126.

[34] Chui J, Poon W, Chan K, Chan A, Buckley T (2005). Nitrite-induced methaemoglobinaemia–aetiology, diagnosis and treatment. Anaesthesia.

[35] Price D. Methemoglobin inducers. Goldfrank’s Toxicologic Emergencies 8th ed New York, NY: McGraw-Hill. 2006:1734–45.

[36] Petróczi A, Naughton DP (2010). Potentially fatal new trend in performance enhancement: a cautionary note on nitrite. J Int Soc Sports Nutr.

[37] Cortazzo JA, Lichtman AD (2014). Methemoglobinemia: a review and recommendations for management. J Cardiothorac Vasc Anesth.

[38] Katabami K, Hayakawa M, Gando S. Severe methemoglobinemia due to sodium nitrite poisoning. Case Rep Emerg Med. 2016. 10.1155/2016/9013816 PMC4987464 27563472

[39] Neth MR, Love JS, Zane Horowitz B, Shertz MD, Sahni R, Daya MR. Fatal sodium nitrite poisoning: key considerations for prehospital providers. Prehosp Emerg Care. 2020:1–7. 10.1080/10903127.2020.1838009 33074043

[40] Hwang C, Yeon SH, Jung J, Na JY. An autopsy case of sodium nitrite-induced methemoglobinemia with various post-mortem analyses. Forensic Sci Med Pathol. 2021. 10.1007/s12024-021-00378-w 33961276

[41] Nitschke P, Stewart F. The Peaceful Pill eHandbook. Blaine (WA): Exit International. 2018.

[42] Byard RW, Winskog C, Heath K (2019). Nitrogen inhalation suicide pacts. Med Sci Law.

[43] Molina Herranz D, García Escudero C, Rite Gracia S, Aguilar de la Red Y, Martínez Nieto J, Izquierdo Álvarez S, Montañés Gracia MA, Recasens V, Hernández Mata CF. CYB5R3 homozygous pathogenic variant as a rare cause of cyanosis in the newborn. Clin Biochem. 2022. 10.1016/j.clinbiochem.2022.01.008 35104462

[44] Kuwada G, Murakami A, Glaser DW, Ingraham SE, Purohit PJ (2022). Mothball Ingestion in the setting of G6PD deficiency causing severe hemolytic anemia, methemoglobinemia, and multiple organ failure in a toddler. Hawaii J Health Soc Welf.

[45] Alyahya B, Alalshaikh A, Sabbahi G, Alnowiser M, Al-Mohawes M. Methylene blue infusion to treat severe dapsone-induced methemoglobinemia in a pediatric patient. Cureus. 2021;13:e18853. 10.7759/cureus.18853 PMC8597667 34804706

[46] Mir WAY, Shrestha DB, Reddy VK, Gaire S, Verda L. A case report of acute transient encephalopathy following a trans-esophageal echocardiography. Cureus. 2021;13:e18580. 10.7759/cureus.18580 PMC8572029 34760423

[47] Chernaya A, Søborg C, Midttun M. Validity of the urinary dipstick test in the diagnosis of urinary tract infections in adults. Dan Med J. 2021;69. 34913433

