# Supplementary material for: Increasing use of sodium nitrite in suicides—an emerging trend
Source: Forensic Sci Med Pathol. 2022 Mar 25;18(3):311–8. doi: 10.1007/s12024-022-00471-8 (PMC9587107; doi:10.1007/s12024-022-00471-8)
Supplement: Supplementary file 1 — Supplementary file1 (DOCX 13 KB) [file 12024_2022_471_MOESM1_ESM.docx]

**Appendix A** Quasi-Poisson regression R output

##

## Call:

## glm(formula = Deaths ~ Year, family = "quasipoisson", data = d)

##

## Deviance Residuals:

## Min 1Q Median 3Q Max

## -0.81190 -0.13224 -0.01294 -0.00125 0.98175

##

## Coefficients:

## Estimate Std. Error t value Pr(>|t|)

## (Intercept) -1978.4616 215.2066 -9.193 3.21e-08 ***

## Year 0.9808 0.1066 9.199 3.18e-08 ***

## ---

## Signif. codes: 0 '***' 0.001 '**' 0.01 '*' 0.05 '.' 0.1 ' ' 1

##

## (Dispersion parameter for quasipoisson family taken to be 0.1091358)

##

## Null deviance: 41.0477 on 19 degrees of freedom

## Residual deviance: 2.3031 on 18 degrees of freedom

## AIC: NA

##

## Number of Fisher Scoring iterations: 8
